# Supplementary material for: Evaluation of Biochemical Serum Markers for the Diagnosis of Polycystic Ovary Syndrome (PCOS) in Obese Women in Kazakhstan: Is Anti-Müllerian Hormone a Potential Marker?
Source: Biomedicines. 2024 Oct 14;12(10):2333. doi: 10.3390/biomedicines12102333 (PMC11504444; doi:10.3390/biomedicines12102333)
Supplement: Supplementary file 1 [file biomedicines-12-02333-s001.zip › biomedicines-3200265-supplementary.pdf]

APPENDIX

Indicator	Control (A) N=46	Obesity		Kruskal-Wallis		*Post-hoc* Dunn's test					
		No PCOS (B) N=67	PCOS (C) N=25			A-B		A-C		B-C	
				H	***p***	Z	***p***	Z	***p***	Z	***p***
Age, years	30.00 (26.00; 33.00)	30.00 (26.00; 34.00)	28.00 (24.75; 30.25)	1.928	0.3814	0.093	>0.9999	1.185	0.7080	1.336	0.5450
BMI, kg/m^2	21.40 (19.73; 23.63)	34.25 (31.05; 38.48)	33.50 (31.38; 36.03)	92.48	<0.0001	9.232	<0.0001	6.670	<0.0001	0.5463	>0.9999
TSH, mIU/mL	2.53 (1.88; 3.30)	2.92 (1.76; 4.31)	2.96 (2.33; 3.92)	5.049	0.0801	2.004	0.1353	1.818	0.2072	0.2849	>0.9999
LH, mIU/mL	5.88 (4.45; 7.08)	4.50 (3.35; 7.30)	9.73 (8.55; 10.79)	24.91	<0.0001	1.315	0.5655	3.682	0.0007	4.978	<0.0001
FSH, mIU/mL	6.49 (5.60; 7.50)	5.82 (4.73; 7.08)	4.44 (3.48; 5.72)	15.34	0.0005	1.156	0.7428	3.857	0.0003	3.145	0.0050
Estradiol, pg/mL	47.14 (31.08; 74.52)	31.46 (19.87; 50.54)	38.83 (23.00; 82.53)	10.30	0.0058	3.187	0.0043	1.124	0.7826	1.412	0.4739
Prolactin, ng/mL	16.26 (12.93; 21.29)	15.62 (10.22; 19.44)	12.91 (10.53; 20.97)	1.144	0.5644	0.7896	>0.9999	1.003	0.9483	0.4177	>0.9999
Progesterone, ng/mL	0.70 (0.39; 0.94)	0.53 (0.38; 0.70)	0.67 (0.49; 1.03)	6.381	0.0412	2.054	0.1200	0.3697	>0.9999	2.070	0.1154
Testosterone, ng/mL	0.19 (0.10; 0.30)	0.21 (0.14; 0.30)	1.42 (1.09; 1.61)	57.69	<0.0001	0.9338	>0.9999	7.167	<0.0001	6.834	<0.0001
Insulin, uIU/mL	7.10 (5.52; 9.69)	17.62 (13.15; 25.05)	15.90 (11.34; 26.04)	58.10	<0.0001	7.298	<0.0001	5.264	<0.0001	0.2848	>0.9999
AMH, ng/mL	4.00 (2.62; 6.86)	1.70 (0.72; 2.64)	9.67 (5.89; 12.65)	58.04	<0.0001	5.132	<0.0001	2.663	0.0232	7.017	<0.0001
Glucose, mmol/L	4.79 (4.59; 5.16)	5.31 (4.92; 5.74)	5.23 (4.99; 5.84)	25.49	<0.0001	4.654	<0.0001	3.886	0.0003	0.3084	>0.9999
Glycated hemoglobin, %	4.54 (3.70; 5.01)	4.08 (3.81; 5.51)	4.30 (3.62; 5.53)	1.829	0.4007	1.216	0.6717	1.082	0.8379	0.1533	>0.9999
Cholesterol, mmol/L	4.19 (3.83; 4.91)	4.89 (4.42; 6.29)	4.58 (4.09; 5.32)	19.70	<0.0001	4.414	<0.0001	1.604	0.3262	1.906	0.1699
HDL, mmol/L	1.50 (1.33; 1.70)	1.21 (1.01; 1.45)	1.07 (0.89; 1.23)	26.81	<0.0001	3.900	0.0003	4.811	<0.0001	1.915	0.1666
LDL, mmol/L	1.98 (1.71; 2.25)	3.12 (2.67; 3.49)	3.02 (2.44; 3.49)	67.85	<0.0001	7.894	<0.0001	5.699	<0.0001	0.4072	>0.9999
Triacylglycerol, mmol/L	0.60 (0.51; 0.81)	1.55 (0.90; 2.53)	1.57 (1.07; 2.17)	51.14	<0.0001	6.747	<0.0001	5.158	<0.0001	0.0282	>0.9999
Adiponectin, ng/mL	16.15 (13.53; 19.40)	8.66 (5.58; 12.67)	7.99 (6.41; 12.25)	47.22	<0.0001	6.571	<0.0001	4.790	<0.0001	0.2904	>0.9999
Resistin, ng/mL	3.74 (1.80; 5.56)	6.54 (4.70; 11.98)	8.09 (3.61; 9.86)	30.95	<0.0001	5.511	<0.0001	3.192	0.0042	1.119	0.7897

Table S1. Statistical parameters including median, lower quartile, upper quartile, Kruskal-Wallis nonparametric test indicators and *post-hoc* Dunn's test indicators. H – test statistic for Kruskal-Wallis nonparametric test; P – p-value; Z – z-score for calculation of p-value in *post-hoc* Dunn's test; P-values<0.05 are considered significant and the corresponding cells are shaded grey. Abbreviations: AMH, anti-Müllerian hormone; BMI, body mass index; FSH, follicle-stimulating hormone; HDL, high density lipoproteins; LDL, low density lipoproteins; LH, luteinizing hormone; PCOS, polycystic ovary syndrome; TSH, thyroid-stimulating hormone
